# Inhibitory effects on L- and N-type calcium channels by a novel Ca_V_β_1_ variant identified in a patient with autism spectrum disorder

**DOI:** 10.1007/s00210-022-02213-7

**Published:** 2022-02-05

**Authors:** Patrick Despang, Sarah Salamon, Alexandra Breitenkamp, Elza Kuzmenkina, Jan Matthes

**Affiliations:** Center of Pharmacology, Institute II, University of Cologne, Gleueler Strasse 24, 50931 Köln, Cologne Germany

**Keywords:** Calcium channels, Patch clamp technique, CACNB1 protein, Autism spectrum disorder

## Abstract

Voltage-gated calcium channel (VGCC) subunits have been genetically associated with autism spectrum disorders (ASD). The properties of the pore-forming VGCC subunit are modulated by auxiliary β-subunits, which exist in four isoforms (Ca_V_β_1-4_). Our previous findings suggested that activation of L-type VGCCs is a common feature of Ca_V_β_2_ subunit mutations found in ASD patients. In the current study, we functionally characterized a novel Ca_V_β_1b_ variant (p.R296C) identified in an ASD patient. We used whole-cell and single-channel patch clamp to study the effect of Ca_V_β_1b_R296C_ on the function of L- and N-type VGCCs. Furthermore, we used co-immunoprecipitation followed by Western blot to evaluate the interaction of the Ca_V_β_1b_-subunits with the RGK-protein Gem. Our data obtained at both, whole-cell and single-channel levels, show that compared to a wild-type Ca_V_β_1b_, the Ca_V_β_1b_R296C_ variant inhibits L- and N-type VGCCs. Interaction with and modulation by the RGK-protein Gem seems to be intact. Our findings indicate functional effects of the Ca_V_β_1b_R296C_ variant differing from that attributed to Ca_V_β_2_ variants found in ASD patients. Further studies have to detail the effects on different VGCC subtypes and on VGCC expression.

**Supplementary Information:**

The online version contains supplementary material available at 10.1007/s00210-022-02213-7.

## Introduction

Autism spectrum disorder (ASD) is a complex neuro-developmental disorder and affects about 1% of the population worldwide (Won et al. 2013). Even though ASD is mainly seen as a genetic disease, the exact etiology is only known in about 20% of the cases, mostly for monogenetic forms of ASD like Timothy syndrome (Splawski et al. 2004; Miles 2011). In ASD, the wide range of genetic findings points to various signaling pathways involved in the manifestation of this disorder (O’Roak et al. 2011; Ebert and Greenberg 2013). A gene pathway analysis with over 2600 patients recognized the “calcium signaling pathway” among the top three pathways involved in ASD (Skafidas et al. 2014). Since the discovery that a single point mutation in the α1-subunit of L-type calcium channels (Ca_V_1.2) causes Timothy syndrome, many other genes encoding voltage-gated calcium channel (VGCC) subunits have been associated with ASD like the genes *CACNA1B-F* encoding pore-forming subunits, *CACNB1* and *CACNB2* encoding auxiliary β-subunits and, *CACNA2D3* and *CACNA2D4* for auxiliary α_2_-δ-subunits (for a comprehensive overview, see Breitenkamp et al. 2015).

Voltage-gated calcium channels (VGCC) are heteromultimeric protein complexes whose composition of pore-forming and auxiliary subunits determines their biophysical properties. High-voltage activated VGCCs are heteromers composed of up to four subunits (Ca_V_α1, Ca_V_β, Ca_V_α_2_-δ, Ca_V_γ) (Buraei and Yang 2010; Campiglio and Flucher 2015). The subunit of particular importance for the regulation of channel function is the Ca_V_β-subunit, with four different isoforms (Ca_V_β_1-4_) involved in the regulation of voltage dependence, channel activity, current kinetics, and also the membrane targeting of functional channels (Meir et al. 2000; Dolphin 2012). Structurally, Ca_V_β-subunits consist of five domains: a variable N-terminus, a conserved Src homology 3 domain (SH3), a conserved but alternatively spliced HOOK domain, a guanylate kinase domain (GK), and a variable C-terminus (Buraei and Yang 2010). With the exception of some rare splice variants, all Ca_V_β-subunits have the same functional structure and show a significant scale of homology and conservation indicating their functional importance (Hibino et al. 2003; Hofmann et al. 2014).

In a previous study, we found three rare missense mutations in highly conserved regions within the susceptibility gene *CACNB2* in ASD-affected patients, all of them within the HOOK-domain (Breitenkamp et al. 2014). In whole-cell recordings, two of the resulting Ca_V_β_2_ mutants (p.G167S, p.S197F) led to a slowed and the third (p.F240L) to an accelerated current inactivation. In a detailed subsequent study, we could show that these mutations and an N-terminal mutation at position 2 (p.V2D) led to a significantly higher single-channel activity compared to channels containing a wild-type (WT) Ca_V_β_2_ (Despang et al. 2020). Mutated and WT Ca_V_β_2_ subunits interacted with the inhibitory RGK-protein Gem but differed in the extent and characteristics of being modulated by Gem. More recently, we described a homozygous N-terminal Ca_V_β_2_ mutant at position 70 (p.R70C) that also showed an increased activity in terms of a reduced inactivation of L-type calcium currents at the whole-cell level (Graziano et al. 2021).

We report here the identification and first electrophysiological characterization of Ca_V_β_1b_R296C_, a novel variant of the β_1_-isoform of VGCC subunits found in an ASD patient. Different genetic studies suggested that similar to Ca_V_β_2_, Ca_V_β_1_ may contribute to ASD. A strong evidence of linkage was found for the chromosomal region 17q11-21 that contains *CACNB1*, the gene encoding Ca_V_β_1_ (Cantor et al. 2005). In a meta-analysis combining affected sib pairs of five genome-wide linkage scans for ASD, no significant chromosomal region for ASD was found (Trikalinos et al. 2006). However, the authors found two chromosomal regions with a suggestive significance, namely, 17p11.2–q12 and 10p12– q11.1, which comprise the genes encoding Ca_V_β_1_ and Ca_V_β_2_, respectively.

Given our previous studies indicating Ca^2+^-current enhancement as a common feature of Ca_V_β_2_ variants found in ASD patients, we characterized the electrophysiological properties of the new Ca_V_β_1_ variant introduced here. Since the different Ca_V_β_2_ variants have shown a kind of “electrophysiological” fingerprint including differential interaction with the RGK-protein Gem, we analyzed Ca_V_β_1_-Gem interaction, too. We chose Ca_V_1.2 as a pore-forming subunit for several reasons: (1) Ca_V_1.2 is expressed in neuronal tissue and has been associated with several neuro-psychiatric diseases (Catterall et al. 2021). (2) A mutant Ca_V_1.2 protein characterized by impaired current inactivation has been shown to underly the Timothy syndrome, a monogenetic “model disease” for ASD (Splawski et al. 2004; Breitenkamp et al. 2015). (3) In our previous studies, we identified functional effects of Ca_V_β_2_ variants when co-expressed with Ca_V_1.2 (Breitenkamp et al. 2014; Despang et al. 2020; Graziano et al. 2021). For the following reasons, Ca_V_2.2 was chosen as a second VGCC pore subunit for our analysis of the Ca_V_β_1b_R296C_ variant: Ca_V_2.2 is expressed predominantly in neurons where most of the central nervous system’s synapses rely on Ca_V_2.2 and Ca_V_2.1 for fast synaptic transmission (Wheeler et al. 1994; Catterall et al. 2021). Three different Ca_V_β subunits associate with the pore-forming subunit Ca_V_2.2, of which Ca_V_β_1b_ is an isoform (Scott et al. 1996; Müller et al. 2010).

## Material and methods

### DNA constructs and site-directed mutagenesis

For functional analysis, the mutation p.R296C was introduced in human Ca_V_β_1b_ (NM_000723) by site-directed mutagenesis (Stratagene QuikChange Kit) and verified by sequencing. EGFP was used as reporter gene, which was co-expressed together with the Ca_V_β-subunit by the bicistronic pIRES2-EGFP vector (Clontech). R296C forward primer 5'-cattgagcgctccaacacatgctccagcct-3'; R296C reverse primer 5'-aggctggagcatgtgttggagcgctcaatg-3'. For co-immunoprecipitation experiments, a hemagglutinin (HA) tag was introduced in the C-terminus of the respective Ca_V_β_1b_ isoform and a Flag tag was introduced at the N-terminus of Gem. Cloning was done by using an in-fusion cloning kit (Takara Bio Europe, Gothenburg, Sweden) according to the manufacturer’s manual and verified by sequencing. EGFP was used as reporter gene for Ca_V_β_1b_ and YFP as reporter for Gem.

### Cell culture and transfection

In brief, HEK-293 cells were grown in Petri dishes in Dulbecco’s modified Eagle’s medium (Gibco Thermo Fisher, Waltham, MA, USA) supplemented with 10% FCS (Biochrom GmbH, Berlin Germany). Cells were routinely passaged twice a week and incubated at 37°C under 5% CO_2_ growth conditions. tsA-201 cells were cultured at 37°C and 5% CO_2_ in a 60-mm-diameter cell culture dish in 4-ml DMEM GlutaMAX medium (Biochrom GmbH, Berlin Germany) with 10% FCS (Biochrom GmbH, Berlin Germany) and penicillin-streptomycin (Sigma-Aldrich, St. Louis, MO, USA). Cells were routinely passaged twice a week. HEK-293 and tsA-201 cells were transfected using standard calcium phosphate method (Koch et al. 2016). For whole-cell and single-channel recordings, HEK-293 cells were transfected with a 1:0.5:1.5 ratio of Ca_V_1.2 (α1c#77, NM_001129843.2) or Ca_V_2.2 (NM_000718.3), either human WT (NM_000723) or mutant Ca_V_β-subunit and human Ca_V_α_2_-δ_1_-subunit (NM_000722.3). For co-immunoprecipitation experiments, tsA-201 cells were transfected with a 1:2 ratio of either human WT or mutant Ca_V_β_1b__HA-subunit and human Flag-Gem.

### Co-immunoprecipitation and Western blot

The Gem and Ca_V_β co-immunoprecipitation experiments were performed as described previously (Despang et al. 2020). In brief, tsA cells were harvested 48–72 h after transfection in ice cold PBS and lysed in lysis buffer (50-mM Tris; 100-mM NaCl; 10-mM EDTA; 0.4% TritonX-100; 0.4% NP-40; pH: 7.5 with HCl). Protease inhibitor cocktail tablets (Sigma-Aldrich) were added immediately before lysing cells. Cell lysates were incubated for 1h at 4°C with rotation and afterwards for 30 min on ice. Cell lysates were then centrifuged for 15 min to remove cell debris. Lysate of 400μl of was incubated with 50μl (500μg)-Pierce™ Protein A/G Magnetic Beads (Thermo Scientific) and 5μl-anti-FLAG-Antibody M2 (Sigma-Aldrich) or 10-μl anti-HA 3F10 (Roche) at 4°C overnight. Beads were washed two times for 15–30 min in lysis buffer, and then proteins were eluted in 2x Laemmli buffer, incubated at 50°C for 10 min and frozen at −20°C. Elutions were separated using SDS-PAGE, followed by immunoblotting with anti-HA antibody (1:1000, Covance) and anti-FLAG antibody M2 (1:1000, Sigma-Aldrich).

### Electrophysiological recordings

Whole-cell recordings were performed as in our previous studies (Breitenkamp et al. 2014; Despang et al. 2020; Graziano et al. 2021). Recordings of EGFP-positive cells were obtained 48–72 h after transfection. Immediately prior to recording, cells kept in 35-mm culture dishes were washed at room temperature (19–23°C) with bath solution. For whole-cell experiments with Ca_V_β_1b_ and α1C (Ca_V_1.2), the bath solution contained (in mM) 10 BaCl_2_, 1 MgCl_2_, 10 HEPES, 65 CsCl, 40 TEA-Cl, 10 Glucose (pH 7.3 with TEA-OH), and the pipette solution (in mM) 140 CsCl, 10 EGTA, 9 HEPES, 1 MgCl_2_, and 4 MgATP (pH 7.3 with CsOH). For whole-cell experiments with Ca_V_β_1b_ and α1B (Ca_V_2.2), the bath solution contained (in mM) 20 BaCl_2_, 1 MgCl_2_, 10 HEPES, 125 NaCl (pH 7.3 with NaOH), and pipette solution (in mM) 135 CsCl, 10 EGTA, 10 HEPES, and 4 MgATP (pH 7.5 with CsOH). Holding potential was −80 mV. Patch pipettes made from borosilicate glass (1.7-mm diameter and 0.283-mm wall thickness, Hilgenberg GmbH, Malsfeld, Germany) were pulled using a Sutter Instrument P-97 horizontal puller and fire-polished using a Narishige MF-83 microforge (Narishige Scientific Instrument Lab, Tokyo, Japan). Pipette resistance was 2–4 MΩ. Using a holding potential of −80 mV, currents were elicited by applying 500, 1000, or 1500 ms test pulses of −40 mV to +50 mV. Currents were sampled at 10 kHz and filtered at 2 kHz. We used the Clampex software pClamp 10 and an Axopatch 200B amplifier (Molecular Devices, Sunnyvale, CA, USA).

Voltage dependence of Ba^2+^ whole-cell current inactivation was determined with a two-pulse protocol (a 2500-ms prepulse followed by a 200-ms test pulse at +10mV). For steady-state inactivation, the relative magnitude of inward current elicited during the second pulse was plotted as a function of the voltage of the conditioning first pulse. Data were analyzed using pClamp10 (Molecular Devices, Sunnyvale, CA, USA) and GraphPad 6 Prism. Currents were filtered at 2 kHz. Data were fitted to a Boltzmann function to obtain the half point (V0.5) and slope factor (dV) for the voltage dependence of inactivation. Fits were performed after subtracting the offset from the peak values of the steady-state inactivation data. Offset was defined as a deviation from zero at the end of a fully inactivating current (at the end of the test pulse at +10mV). For voltage dependence of activation, data were fitted by combined Ohm and Boltzmann relation $\mathrm{I}\left(\mathrm{V}\right)=\left(\mathrm{V}\hbox{--} \mathrm{VR}\right)\ \mathrm{x}\frac{G_{max}}{\left(1+\exp \frac{\left({V}_{0.5}-V\right)}{dV}\right)}$ according to Karmazinova and Lacinova (Karmažínová and Lacinová 2010).

Single-channel recordings were performed at room temperature (19–23°C) using EGFP-positive cells 48–72 h after transfection. The bath solution contained (in mM) 120 K-glutamate, 25 KCl, 2 MgCl_2_, 10 HEPES, 1 CaCl_2_, 1 Na-ATP, and 2 EGTA (pH 7.4 with KOH). Patch pipettes were made from borosilicate glass (1.7-mm diameter and 0.283-mm wall thickness, Hilgenberg GmbH, Malsfeld, Germany) using a Narishige PP-83 vertical puller, Sylgard coated and fire-polished using a Narishige MF-83 microforge (Narishige Scientific Instrument Lab, Tokyo, Japan). Pipette resistance was 5–7 MΩ. Pipettes were filled using an internal solution containing (in mM) 110 BaCl_2_ and 10 HEPES (pH 7.3 with TEA-OH). Single calcium channels were recorded in the cell-attached configuration (depolarizing test pulses of 150 ms at 1.67 Hz from −20mV to +20 mV in 10 mV steps from a holding potential of −100mV). An Axopatch 1D amplifier with pClamp 5.5 software (both from Axon Instruments, Foster City, CA, USA) was used for pulse generation, data acquisition (10 kHz), and filtering (2 kHz, −3 dB, 4-pole Bessel). Experiments were analyzed when channel activity persisted > 180 sweeps using pClamp 10 software (Molecular Devices).

### Data analysis

Most experimental data are shown as raw currents, i.e., without capacity or leak subtraction. In a few experiments, for reasons of clarity, the capacitive transients were removed off-line. For calculation of the activation, the leak-current component was obtained from hyperpolarizing voltage steps from V_h_ and fitted using linear regression. The extrapolated leak current was then subtracted from the current-voltage (I-V) plot before estimation of the reversal potential. For single-channel analysis, leak and capacity currents were digitally subtracted using pClamp 10 software (Molecular Devices). Channel openings and closures were determined using the half-height criterion. The availability (fraction of active sweeps, F_active_), open probability (P_open_), and mean open time were analyzed for single-channel as well as multi-channel patches. For the latter, these parameters were corrected for the number of channels in the patch (Herzig et al. 2007).

### Statistical analysis

Electrophysiological data were obtained in experiments revealed from > 3 individual transfections per plasmid composition and are shown as mean ± SEM. Experiments with WT and mutant were performed on the same day. Data were analyzed using unpaired two-sided Student *t* tests. Differences were considered statistically significant if *p* < 0.05.

### Ethics approval

The local ethics committee approved the analyses (ref. No. 04-223) as described earlier (Breitenkamp et al. 2014).

## Results

### Description and genetic assessment of the Ca_V_β_1b_ mutation

We refer to the same probes analyzed previously (Breitenkamp et al. 2014). In a mutation screening involving 155 ASD patients, we found a rare missense mutation in the candidate gene *CACNB1* leading to an arginine-to-cysteine exchange (p.R296C) in the highly conserved GK-domain of the Ca_V_β_1_ subunit (NP_000714.3). The variant was found in a male patient (AGRE ID: AU09935301), but in none of 259 matching controls. The mutation was present in all splice variants of the patient’s *CACNB1* gene. No other pathogenic or suspicious mutations known to contribute to neurodevelopmental disorders were found. No data on the presence of the *CACNB1* mutation in his parents are available, but they were not diagnosed with ASD.

The variant (chr17:37340296G>A; GRCh37) is listed in dbSNP (rs746198242) and in the gnomAD and ExAC databases with an allele frequency of 1.6 x 10^-5^ and 1.7 x 10^-5^, respectively. The here presented *CACNB1* variant p.R296C is not listed in ClinVar (https://www.ncbi.nlm.nih.gov/clinvar) and has not been linked to ASD so far. According to the web-based program “PolyPhen2” (Adzhubei et al. 2010), the p.R296C missense variant is classified as damaging with 100% probability. According to standards and guidelines for the international interpretation of sequence variants (Richards et al. 2015), the rare frequency, the location of the variant in an essential and highly conserved protein domain, and the predictions render this variant a “variant of uncertain significance” (class 3, due to AGMC criteria PM2, PM1 and PP3). In the following, we tested for putative functional effects in electrophysiological patch-clamp experiments. Unlike Ca_V_β_1a_ and Ca_V_β_1c_, the splice variant Ca_V_β_1b_ is expressed in the brain and is upregulated during ontogenesis (Buraei and Yang 2010). Therefore, we subcloned the mutation into the splice form Ca_V_β_1b_ (NM_000723).

### Functional characterization of the Ca_V_β_1b_ variant

#### Whole-cell recordings

**Fig. 1:**
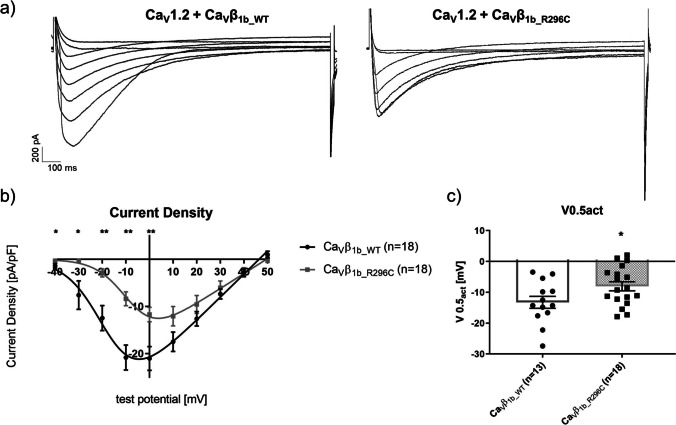
Whole-cell currents mediated by Ca_V_1.2 co-expressed with Ca_V_α_2_-δ_1_ and either the wild-type (Ca_V_β_1b_WT_) or mutant (Ca_V_β_1b_R296C_) form of Ca_V_β_1b_. (**a**) Exemplary traces of whole-cell recordings with either Ca_V_β_1b_WT_ (left) or Ca_V_β_1b_R296C_ (right). (**b**) IV-relationships of Ca_V_β_1b_WT_ (*n*=18) and Ca_V_β_1b_R296C_ (*n*=18). Starting from a holding potential of −80 mV, currents were elicited at test potentials from −40 to +50 mV in 10 mV increments using 10 mM Ba^2+^ as charge carrier. (**c**) Test potential of the half-maximal activation (V0.5_act_). Data were obtained using patch-clamp recordings in the whole-cell configuration. *,**: *p*<0.05 and *p*<0.01 in Student’s *t* tests

**Fig. 2:**
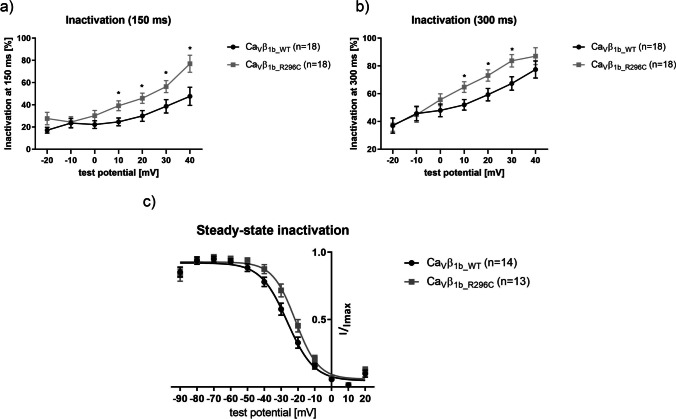
Time-dependent inactivation behavior of Ca_V_1.2 co-expressed with Ca_V_α_2_-δ_1_ and either the wild-type (Ca_V_β_1b_WT_; *n*=18) or mutant (Ca_V_β_1b_R296C_; *n*=18) form of Ca_V_β_1b_, analyzed as the fraction of peak current inactivated after 150 ms (**a**) or 300 ms (**b**) of depolarization. (**c**) Voltage-dependent steady-state inactivation behavior of Ca_V_β_1b_WT_ (*n*=14) compared to Ca_V_β_1b_R296C_ (*n*=13). Data were obtained using patch-clamp recordings in the whole-cell configuration. *: *p*<0.05 in Student’s *t* tests

**Fig. 3:**
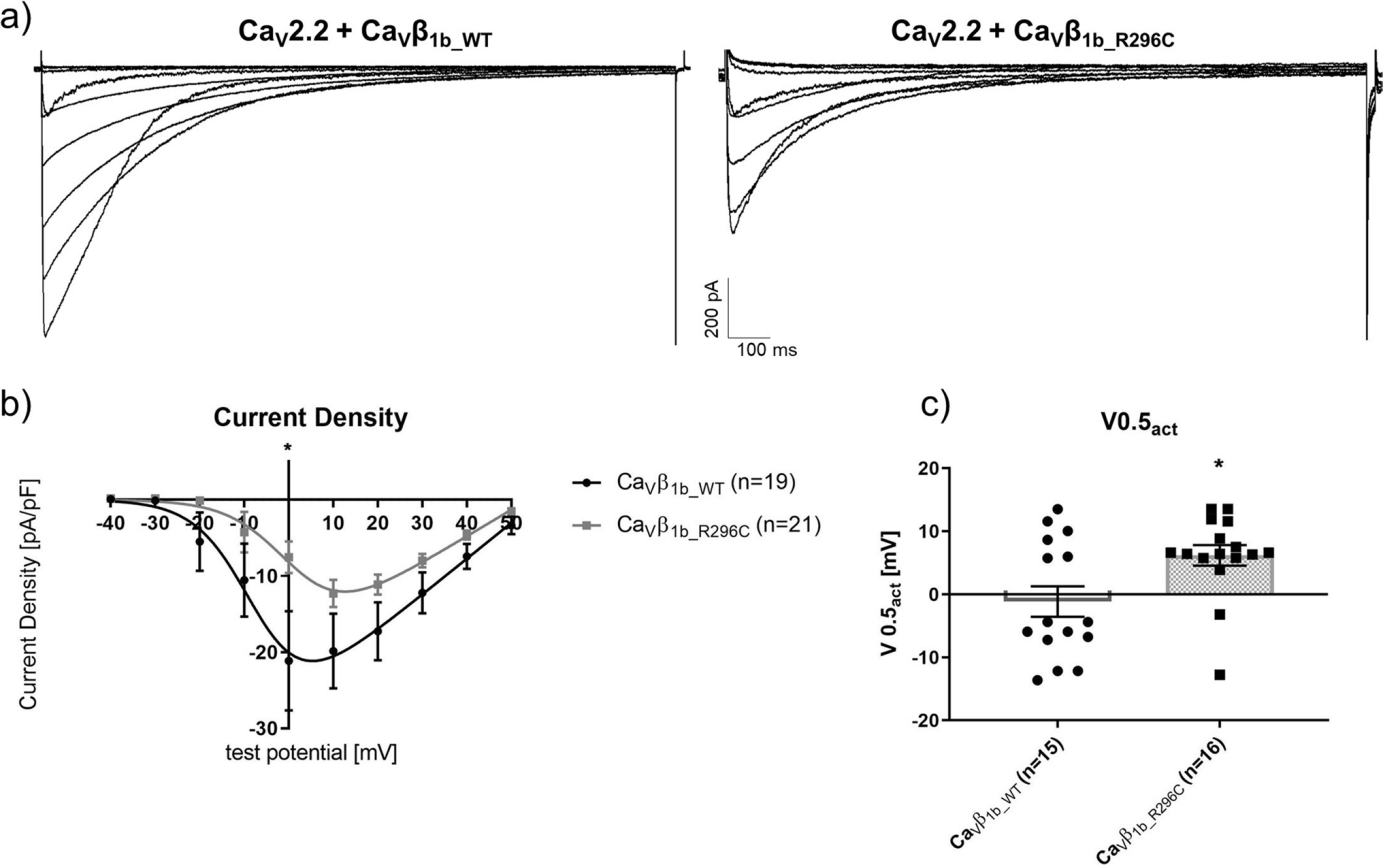
Whole-cell currents mediated by Ca_V_2.2 co-expressed with Ca_V_α_2_-δ_1_ and either the wildtype (Ca_V_β_1b_WT_) or mutant (Ca_V_β_1b_R296C_) form of Ca_V_β_1b_. (**a**) Exemplary traces of whole-cell recordings with either Ca_V_β_1b_WT_ (left) or Ca_V_β_1b_R296C_ (right). (**b**) IV-relationships of Ca_V_β_1b_WT_ (n=19) and Ca_V_β_1b_R296C_ (n=21). Starting from a holding potential of -80 mV, currents were elicited at test potentials from -40 to +50 mV in 10 mV increments using 20 mM Ba^2+^ as charge carrier. **(c)** Test potential of the half-maximal activation (V0.5_act_). Data were obtained using patch-clamp recordings in the whole-cell configuration. *: p<0.05 in Student’s *t* tests

When co-expressing Ca_V_1.2 with the Ca_V_β_1b_R296C_ mutant, whole-cell current density was decreased compared to the wild-type Ca_V_β_1b_ (e.g., 21.0 ± 2.5 vs. 11.7 ± 1.6 pA/pF at 0 mV, *p*=0.003; Figs. 1a and b) along with a shift in the half-maximal activation potential V0.5_act_ to more positive values (−13.3 ± 1.9 vs. −8.0 ± 1.5 mV, *p*=0.038; Fig. 1c). Furthermore, time-dependent inactivation was increased at 150 ms (e.g., 25 ± 4 vs. 39 ± 4 % at +10 mV, *p*=0.015) and 300 ms (e.g. 52 ± 4 vs. 65 ± 4 % at +10 mV, *p*=0.03) (Figs. 2a and b). No statistically significant differences were found at 500 ms, 1000 ms, and 1500 ms (not shown). Analysis of steady-state inactivation revealed a non-significant (*p*=0.071) shift of Ca_V_β_1b_R296C_ to more positive potentials compared to Ca_V_β_1b_WT_ (Fig. 2c). Similar to Ca_V_1.2, the current density was decreased when Ca_V_2.2 was co-expressed with Ca_V_β_1b_R296C_ compared to Ca_V_β_1b_WT_, although the difference was only statistically significant at 0 mV (*p*=0.045). V0.5_act_ was shifted to more positive values by Ca_V_β_1b_R296C_ (−1.1 ± 2.4 vs. 6.2 ± 1.6 mV, *p*=0.017; Fig. 3c). No difference in time-dependent inactivation or steady-state inactivation was found between mutant and wild-type Ca_V_β_1b_ subunits (not shown).

#### Single-channel recordings

**Fig. 4:**
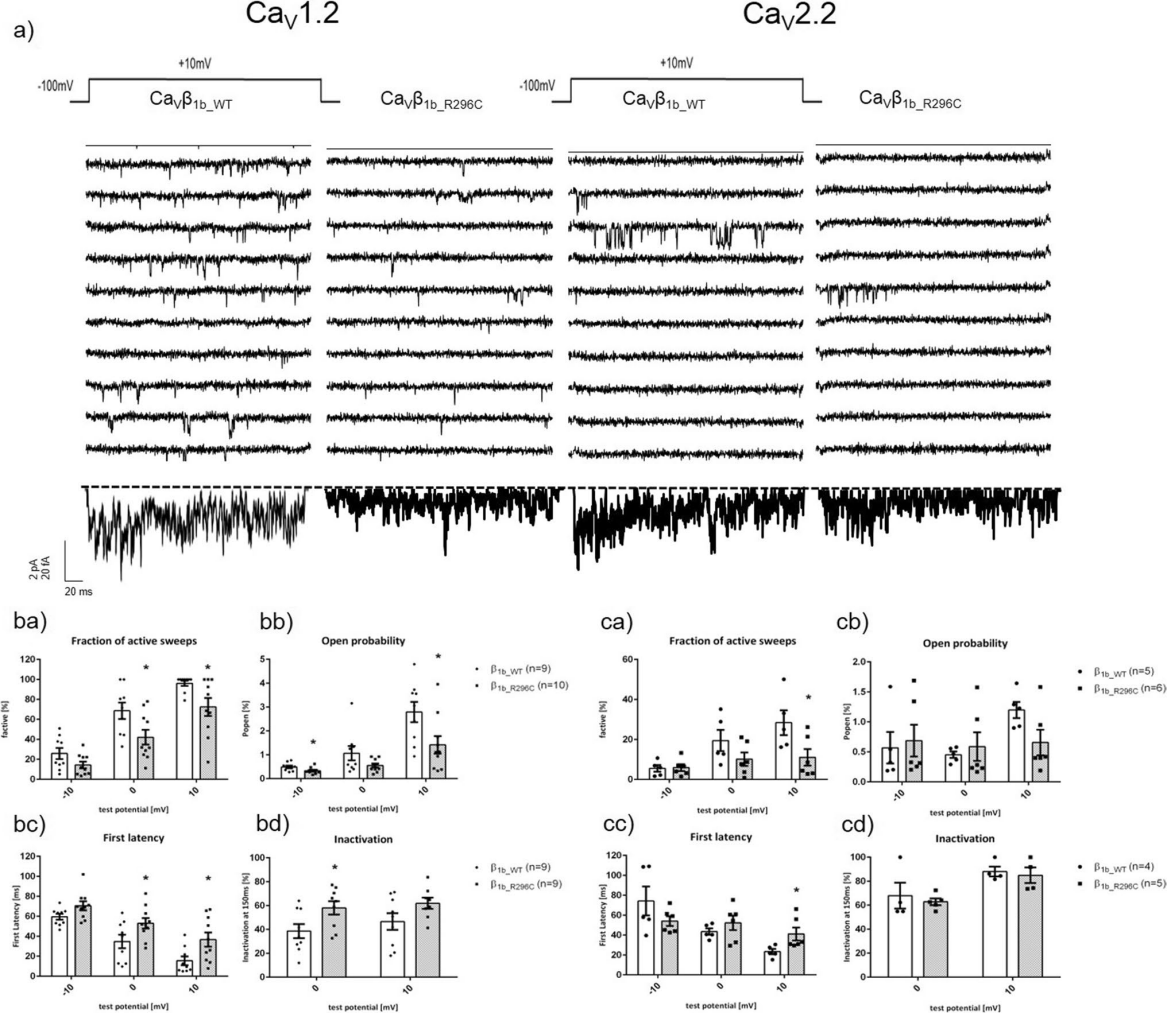
Gating of single L-type (Ca_V_1.2, left) and N-type (Ca_V_2.2, right) channels in the presence of a wildtype (Ca_V_β_1b_WT_) or the mutant (Ca_V_β_1b_R296C_) form of Ca_V_β_1b_. (**a**) Exemplary traces. Top: pulse protocol (150 ms duration, holding potential of -100 mV, test pulse to +10 mV). Middle: ten consecutive traces for each channel complex. Bottom: ensemble average currents of the respective experiments. (**b, c**) Single-channel gating parameters of human Ca_V_1.2 (**ba-bd**; *n*=9–10) or Ca_V_2.2 (**ca-cd**; *n*=5–6) transiently expressed in HEK-293 cells and co-transfected with Ca_V_β_1b_WT_ or Ca_V_β_1b_R296C_ and a human Ca_V_α_2_-δ_1_. Statistical analyses revealed a significant decrease in the fraction of active sweeps and an increase in first latency in the presence of Ca_V_β_1b_R296C_ (**ba, bc, ca, cc**). The reduction of the open probability was statistically significant with Ca_V_1.2 (**bb**) but slightly missed statistical significance with Ca_V_2.2 (**cb**; *p*=0.07). Inactivation was significantly increased when co-expressing Ca_V_β_1b_R296C_ together with Ca_V_1.2 (**bd**), but unaffected with Ca_V_2.2 (**cd**). Data were obtained using patch-clamp recordings in the cell-attached configuration using 110mM Ba^2+^ as charge carrier. *: *p*<0.05 in Student’s *t* tests

At the single-channel level, Ca_V_β_1b_R296C_ showed statistically significant inhibitory effects on both Ca_V_1.2 and Ca_V_2.2. Fraction of active sweeps (F_active_) and open probability (P_open_) of Ca_V_1.2 were significantly reduced when co-expressing Ca_V_β_1b_R296C_ instead of the Ca_V_β_1b_WT_ (Figs. 4 ba and bb). The latency until a first opening and the extent of inactivation were increased (Figs. 4 bc and bd). Similar effects of Ca_V_β_1b_R296C_ were seen on the activity of single Ca_V_2.2 reaching statistical significance for fraction of active sweeps and first latency (Figs. 4 ca and cc).

#### Effects of Gem co-expression on whole-cell and single-channel activity

**Fig. 5:**
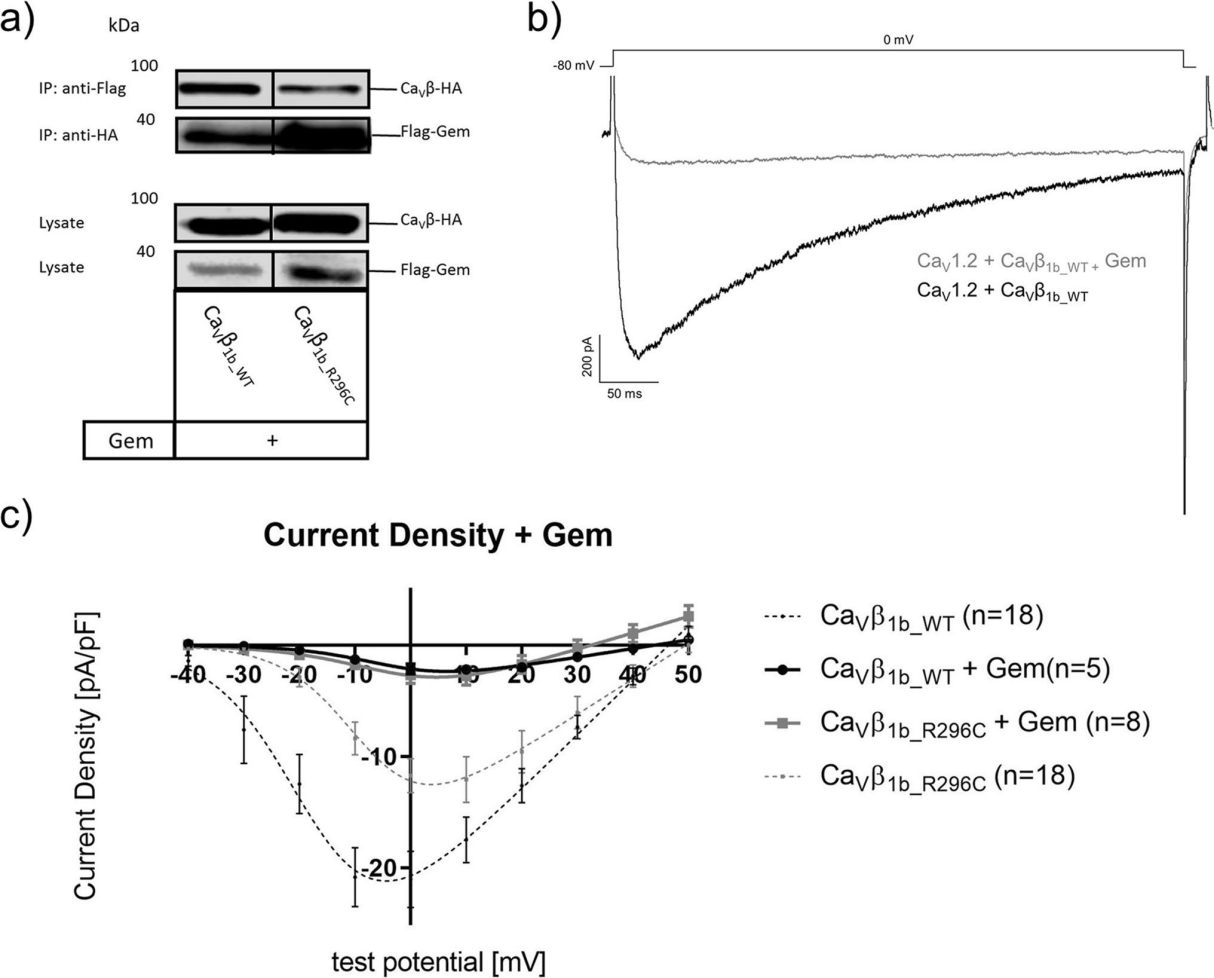
Ca_V_β-Gem interaction and Gem effects on Ca_V_1.2-mediated whole-cell currents in the presence of either the wild-type (Ca_V_β_1b_WT_) or mutant (Ca_V_β_1b_R296C_) form of Ca_V_β_1b_. (**a**) Gem binds to both, Ca_V_β_1b_WT_ and Ca_V_β_1b_R296C_. For co-immunoprecipitation experiments, tsA-201 cells were co-transfected with cDNA of Flag-tagged Gem and HA-tagged Ca_V_β_1b_ subunits as indicated. Flag-tagged Gem proteins were immunoprecipitated (IP) and bound Ca_V_β_1b_ protein was detected by Western-blot. Interaction was confirmed by reciprocal immunoprecipitation. Cell lysates were blotted with a HA- or Flag-antibody to verify Ca_V_β_1b_ and Gem expression, respectively. All experiments were repeated at least three times. (**b**) Exemplary traces of whole-cell recordings in HEK293 cells with expression of Ca_V_β_1b_WT_ at a test potential of 0 mV. Exemplary traces are shown as overlay to better visualize the reduction of currents in the presence of Gem. (**c**) IV-relationships in Gem-transfected HEK293 cells with expression of either Ca_V_β_1b_WT_ or Ca_V_β_1b_R296C_ (solid curves). For comparison, IV-relationships without co-transfection of Gem is shown as dashed curves (taken from Fig. 1). Starting from a holding potential of −80 mV, currents were elicited at test potentials from −40 to +50 mV in 10 mV increments using 10 mM Ba^2+^ as charge carrier. Data were obtained using patch-clamp recordings in the whole-cell configuration

**Fig. 6:**
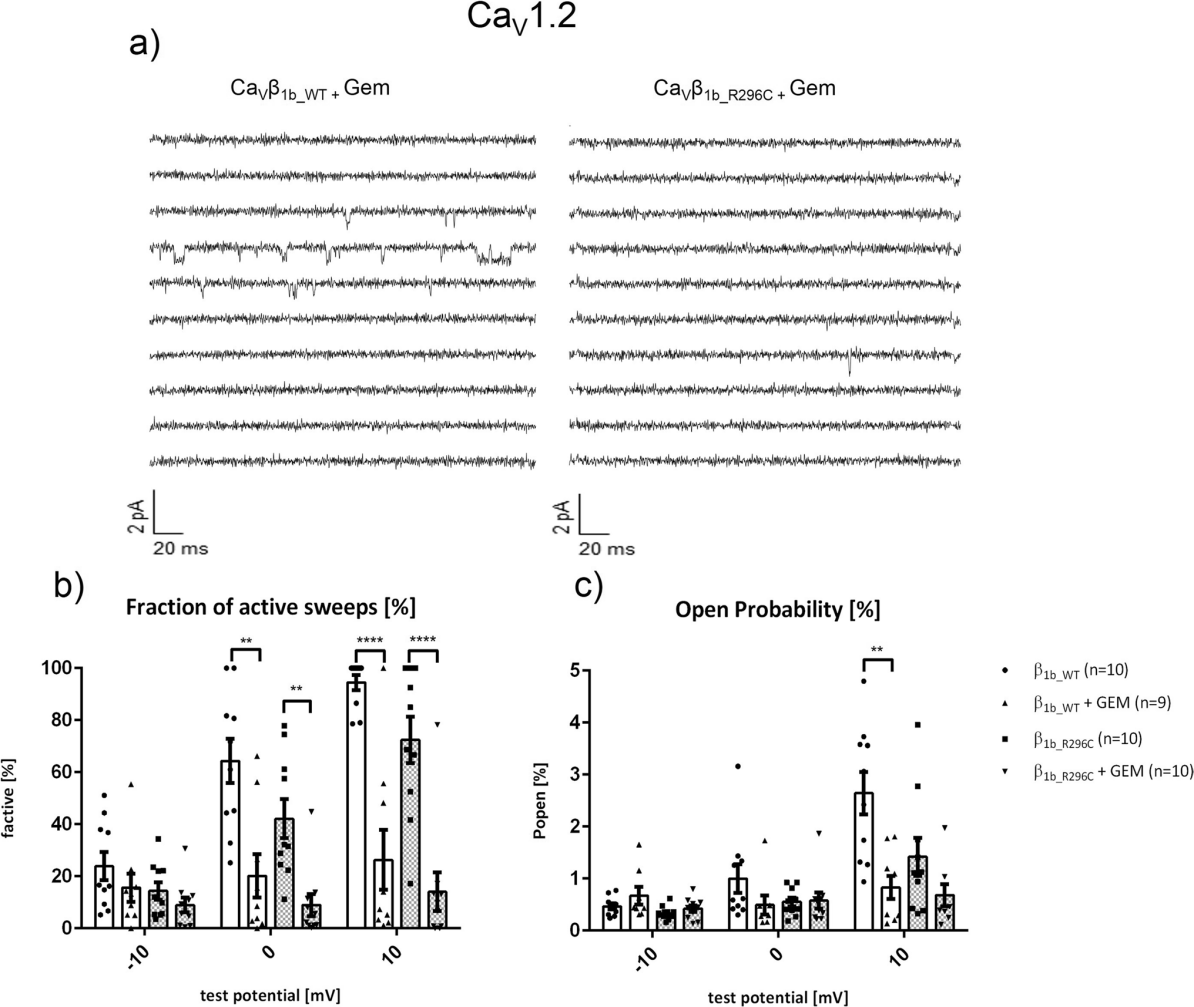
Effects of Gem on single-channel behavior of L-type (Ca_V_1.2) calcium channels in the presence of either the wild-type (Ca_V_β_1b_WT_) or the mutant (Ca_V_β_1b_R296C_) form of Ca_V_β_1b_. (**a**) Ten representative consecutive traces obtained at a test potential of +10 mV in Gem-transfected HEK293 cells expressing either Ca_V_β_1b_WT_ (left) or Ca_V_β_1b_R296C_ (right). Fraction of active sweeps (**b**) and open probability (**c**) indicate similarly reduced activity of single Ca_V_1.2 when Gem was co-expressed together with either Ca_V_β_1b_WT_ or Ca_V_β_1b_R296C_. Number of experiments are indicated in the figure. Asterisks indicate *p*<0.05 in Student’s *t* tests. Data were obtained using patch-clamp recordings in the cell-attached configuration (charge carrier: 110 mM Ba^2+^)

Gem is an RGK-protein that interacts with auxiliary Ca_V_β subunits and inhibits VGCCs (Yang and Colecraft 2013). As in our previous study (Despang et al. 2020), we analyzed the interaction of Gem with the mutated Ca_V_β_1b_ and the effects on Ca_V_1.2-mediated Ba^2+^ currents. Similar to our previous findings with Ca_V_β_2d_, co-immunoprecipitation experiments showed an interaction of Gem with both mutated and wild-type Ca_V_β_1b_ subunits (Fig. 5a). In whole-cell experiments, we saw an almost complete inhibition of Ba^2+^ currents compared to experiments without Gem transfection (Figs. 5b and c). Gem reduced whole-cell currents similarly in the presence of Ca_V_β_1b_WT_ or Ca_V_β_1b_R296C_ (Fig. 5c). Corresponding to our findings at the whole-cell level, we found Gem to reduce single-channel activity, e.g., by lowering the fraction of active sweeps or open probability (Fig. 6).

## Discussion

In the last years, the evidence for the involvement of VGCCs in ASD has been growing and actually for most VGCC genes evidence for an association with ASD was found (Breitenkamp et al. 2015). Our group focuses on auxiliary Ca_V_β subunits of VGCCs and their putative contribution to this complex-genetic disease. To our knowledge, this is the first report of a mutation in *CACNB1* identified in an ASD patient. We previously described different mutations of the Ca_V_β_2_ isoform which led to an increase of Ca_V_1.2 activity both at the single-channel and the whole-cell level (Breitenkamp et al. 2014; Despang et al. 2020). In a more recent study, we could show that another mutation in *CACNB2* led to a reduced inactivation of whole-cell currents, which also indicates increased Ca_V_1.2 activity (Graziano et al. 2021). Taken together, the increase in Ca_V_1.2 activity appears to be a common feature among Ca_V_β_2_ variants found in ASD patients. In contrast, the *CACNB1* mutation described here results in a Ca_V_β_1_ variant that, compared to a wild-type Ca_V_β_1_, decreases current density and shifts half-maximal activation to more positive potentials when co-expressed with Ca_V_1.2 or Ca_V_2.2 and it increases inactivation of Ca_V_1.2. These findings are consistent with the reduced single-channel activity of both, L-type (Ca_V_1.2) and N-type (Ca_V_2.2) VGCCs. Ca_V_β_1b_R296C_ thus can be considered a loss-of-function mutation.

To our knowledge, reduced single-channel activity has not been described in the context of ASD-associated VGCC variants so far. Even more, the only (electrophysiological) loss-of-function variant of Ca_V_β_1_ we are aware of was described in the context of Brugada syndrome, a cardiac disease associated with arrhythmia (Antzelevitch et al. 2007). Interestingly, there are Ca_V_β_2_ variants associated with Brugada syndrome that lead to decreased whole-cell currents (Ca_V_β_2b_S481L_) or accelerated inactivation of Ca_V_1.2 (Ca_V_β_2b_T11I_) (Antzelevitch et al. 2007; Cordeiro et al. 2009). The affected Ca_V_β_2b_ subunit is the most abundant Ca_V_β subunit in the heart but is also expressed in the brain (Hullin et al. 2007; Buraei and Yang 2010). Nevertheless, the patients described in the context of Brugada syndrome did not show a neurodevelopmental phenotype. Like Ca_V_β_2b_, Ca_V_β_1b_ is one of several Ca_V_β subunits expressed in the brain (in addition to, e.g., Ca_V_β_2a,e_, Ca_V_β_3_, and Ca_V_β_4_) (Buraei and Yang 2010). Similar to Ca_V_β_2_ variants found in ASD patients (Despang et al. 2020), the Ca_V_β_1b_ variant described here shows a heterozygous expression profile. It has been shown that different Ca_V_β isoforms can compete for the interaction with the Ca_V_1.2 subunit by this modulating VGCC activity dynamically on a minute timescale (Jangsangthong et al. 2011). In another study, co-expression of Ca_V_β_2a_ and Ca_V_β_3_ resulted in two biophysical distinct channel populations (Jones et al. 1998). Thus, we cannot exclude that the inhibitory effects of the Ca_V_β_1b_R296C_ variant observed in the current study might be (partially) compensated by other Ca_V_β isoforms and/or splice variants in a more physiological expression system.

The mutation described in this study lies within the GK-domain of Ca_V_β_1_, a region well established as a protein-protein interaction site, especially for the intramolecular interactions with the SH3-domain critically involved in the function of Ca_V_β subunits (Buraei and Yang 2010). Alterations affecting these domains have been shown to change the effects of Ca_V_β subunits on VGCC gating (Chen et al. 2009). Furthermore, GK-GK domain or SH3-SH3 domain interactions are involved in Ca_V_β dimerization (Lao et al. 2010; Miranda-Laferte et al. 2011). Of note, the formation of SH3-SH3 dimers is probably mediated by a cysteine residue (Miranda-Laferte et al. 2011). Whether the introduction of a cysteine in the GK-domain of our Ca_V_β_1b_R296C_ variant has a similar effect should be investigated in future studies. According to the web-based prediction program DiANNA 1.1 (Ferrè and Clote 2005), the exchange from arginine to cysteine results in an additional disulfide bond and by this a potentially misfolded tertiary structure of the mutant compared to the wild-type Ca_V_β_1b_.

It has been shown that the Ca_V_β_1b_ subunit prevents Ca_V_1.2 from degradation and facilitates its export from the endoplasmic reticulum (Altier et al. 2011). Similarly, it prevented Ca_V_2.2 from proteasomal degradation (Page et al. 2016). In this context, a recent study could show that the Ca_V_β subunit is imperative for the anterograde transport of Ca_V_1.2 channels to the plasma membrane, while the retrograde transport seems to be Ca_V_β independent (Conrad et al. 2021). Thus, we cannot exclude that beneath single-channel activity, VGCC expression might be affected by the examined Ca_V_β variant. RGK-proteins in general are known VGCC inhibitors by directly inhibiting the activity of VGCCs or altering number of channels at the membrane (Buraei et al. 2015). While some of the RKG-proteins can regulate VGCC activity by directly interacting with the pore-forming subunit of VGCC complexes, Gem utilizes a solely Ca_V_β-dependent mode of action (Puckerin et al. 2016, 2018). In the present study, co-expression of the RGK-protein Gem similarly reduced whole-cell currents mediated by VGCCs co-expressed with either Ca_V_β_1b_R296C_ or Ca_V_β_1b_WT_. In our previous study, Ca_V_β_2_ variants found in ASD patients showed a differential modulation of Ca_V_1.2 compared to each other and to the respective wild-type Ca_V_β_2_ (Despang et al. 2020). Of note, this was also seen in differences between Gem-mediated inhibitory effects. RGK-proteins have been proposed to have potentials as therapeutic tools for a differential and specific modulation of VGCCs (Colecraft 2020). It is tempting to speculate that RGK proteins might be a tool to differentially compensate for changes of VGCC-mediated currents that are due to mutated Ca_V_β subunits.

The alterations caused by co-expressing the Ca_V_β_1b_R296C_ variant appear to be less pronounced on Ca_V_2.2 compared to Ca_V_1.2, both in respect to whole-cell currents and single-channel gating. Differences between modulation of Ca_V_1 and Ca_V_2 VGCCs by Ca_V_β subunits have already been described (Buraei and Yang 2010). Future studies should address this in more detail.

## Conclusion

While evidence for *CACNB2* involvement in ASD is growing, this is the first report of a *CACNB1* variant found in an ASD patient that shows functional consequences, at least at the molecular level. Interestingly, the effects of the Ca_V_β_1b_R296C_ described here are opposite to those described for Ca_V_β_2_ variants previously, i.e. an inhibitory instead of a stimulatory effect compared to the respective WT Ca_V_β isoform. This might indicate differential roles of different Ca_V_β isoforms in the context of ASD. However, the electrophysiological effects we have described at the molecular and cellular level need further investigation in models of more pathophysiological relevance.

## Supplementary Information

ESM 1 (XLSX 75 kb)
